# Evaluation of an assistance system supporting older pedestrians’ road crossing in virtual reality and in a real-world field test

**DOI:** 10.3389/fpsyg.2022.966096

**Published:** 2022-12-20

**Authors:** Rebecca Wiczorek, Janna Protzak

**Affiliations:** Research group FANS, Department of Psychology and Ergonomics, Technische Universität Berlin, Berlin, Germany

**Keywords:** older pedestrians, assistance system, road crossing, virtual reality, field study

## Abstract

Older pedestrians are at a high risk of becoming victims of car accidents because they tend not to pay sufficient attention to upcoming traffic. Within our research project, an assistance system for older pedestrians has been developed. It detects the street and communicates with the users through a vibrotactile interface. Two evaluation studies have been carried out in order to understand the potential benefits and drawbacks of the developed assistance system. One study was conducted in a virtual environment (VR) with 23 participants, aged 65+. The other experiment was a field test in a real street environment with 26 participants, aged 65+. Objective dependent variables in both experiments were checking for traffic (operationalized *via* head tracking) and stopping in front of the street (VR study), i.e., approaching time (field test). Workload and acceptance served as subjective dependent variables. Analysis of the VR experiment showed significantly more head rotation with the assistance system than without it, as well as significantly more with cars than without cars. The same was true for the frequency of stopping. No significant difference was found concerning workload. With regard to acceptance, the majority of participants indicated that the system was supportive and able to reduce risks in traffic. In the field test, results for head rotation confirmed the findings of the VR study. Analysis showed a marginally significant higher head rotation frequency with the alarm system than without, and significantly different patterns of checking for traffic at marked and unmarked crossings. However, unlike in the VR study, no differences were found in approaching time with and without the assistance system. Approaching time was slower at marked crossings. No difference was found with regard to workload, meaning the use of the assistance system did not increase the subjectively perceived workload of participants. Analysis of the acceptance questionnaire showed a positive attachment to the assistance system. However, most reported that they did not experience any advantage from the use of the system, and expressed no intention to buy such a system for themselves.

## Introduction

Older pedestrians are at a high risk of becoming victims of car crashes, as official statistics show [Statistisches Bundesamt (Destatis), 2020, 2021]. In 2019, around 20% of the victims of car crashes in Germany were 60 years and older. Moreover, their risk of dying as a result of an accident is three times higher compared to younger victims, which is due to their higher fragility. Seventy-eight percent of the accidents involving older pedestrians were caused by the older pedestrians. The official statistics also indicate an important reason for older pedestrians’ higher involvement in such accidents: In more than half of the cases, the older pedestrians did not pay sufficient attention to the upcoming traffic.

Prior research supports the conclusion that insufficient attention paid to traffic is prevalent and offers some explanations. One important reason for a lack of attention to traffic is engagement in parallel visual tasks. Two laboratory experiments (Zito et al., 2015; Tapiro et al., 2016) indicate higher frequencies of checking the ground for obstacles in older compared to younger pedestrians. Avineri et al. (2012) found a correlation between this ground checking behavior and a fear of falling, which increases with age (Tinetti et al., 1994; Schott, 2008). Further, Wiczorek and Protzak (2022) show the negative impact of visual and cognitive tasks on hazard perception in a road crossing simulation.

Another reason for insufficient attention paid to upcoming traffic is engagement in parallel motor tasks, namely in walking. An observation study and a photo-based questionnaire (Wiczorek et al., 2016) suggests that both younger and older pedestrians do not usually stop in front of a street to check for traffic. Instead, they tend to keep walking and move their heads to check for traffic while approaching the street. In an EEG-experiment using a dual-task paradigm of combined real over ground walking and visual signal detection, it was found that the number of missed visual signals did significantly increase from standing to walking, but only for older participants (Protzak et al., 2021).

Within the research group FANS, an assistance system has been developed with the aim to support older pedestrians’ road crossing. The system was developed to detect the street rather than approaching cars because the latter is not possible yet. In order to detect an approaching car, the sensors used for the system need a free field. In a lot of urban street environments, this is not possible due to obstacles such as trees, poles and, most importantly, parked cars. If, in the future, cars are capable of car-to-car communication or, in this case, car-to-device communication, detection of approaching cars will be a helpful way to increase the safety of pedestrians.

However, the aim of the current system is to detect the street and to remind the users to refrain from any parallel activities, namely, to stop checking the floor and to stop walking. Instead, they should focus their whole attention on the traffic. Detection of the street has been realized through a combination of sensor fusion and machine learning (Qureshi et al., 2018; Qureshi and Wizcorek, 2019). The system, which is mounted to a walking frame, detects the curb stone using a webcam in addition to an infrared-based LEDDAR sensor. The detection rate has been optimized using CNN algorithms up to an efficiency of more than 99%. The system was trained to detect only the kerbstone between the pathway and the street when approaching the street, but not the kerbstone between the street and the pathway on the other side of the road.

Three different interfaces (auditory, thermotactile, and vibrotactile) have been investigated in a laboratory experiment with older participants (Wiczorek, under review).^1^ The one that was both efficient and had a high acceptance rate by the older people was the vibrotactile interface. The vibrotactile interface was realized through vibrating cuffs, worn at the upper arms. This placement was chosen to direct users’ attention as close to the traffic as possible when their first reaction is a shift of attention to the application of stimulus (Bradley, 2009).

**Figure 1:**
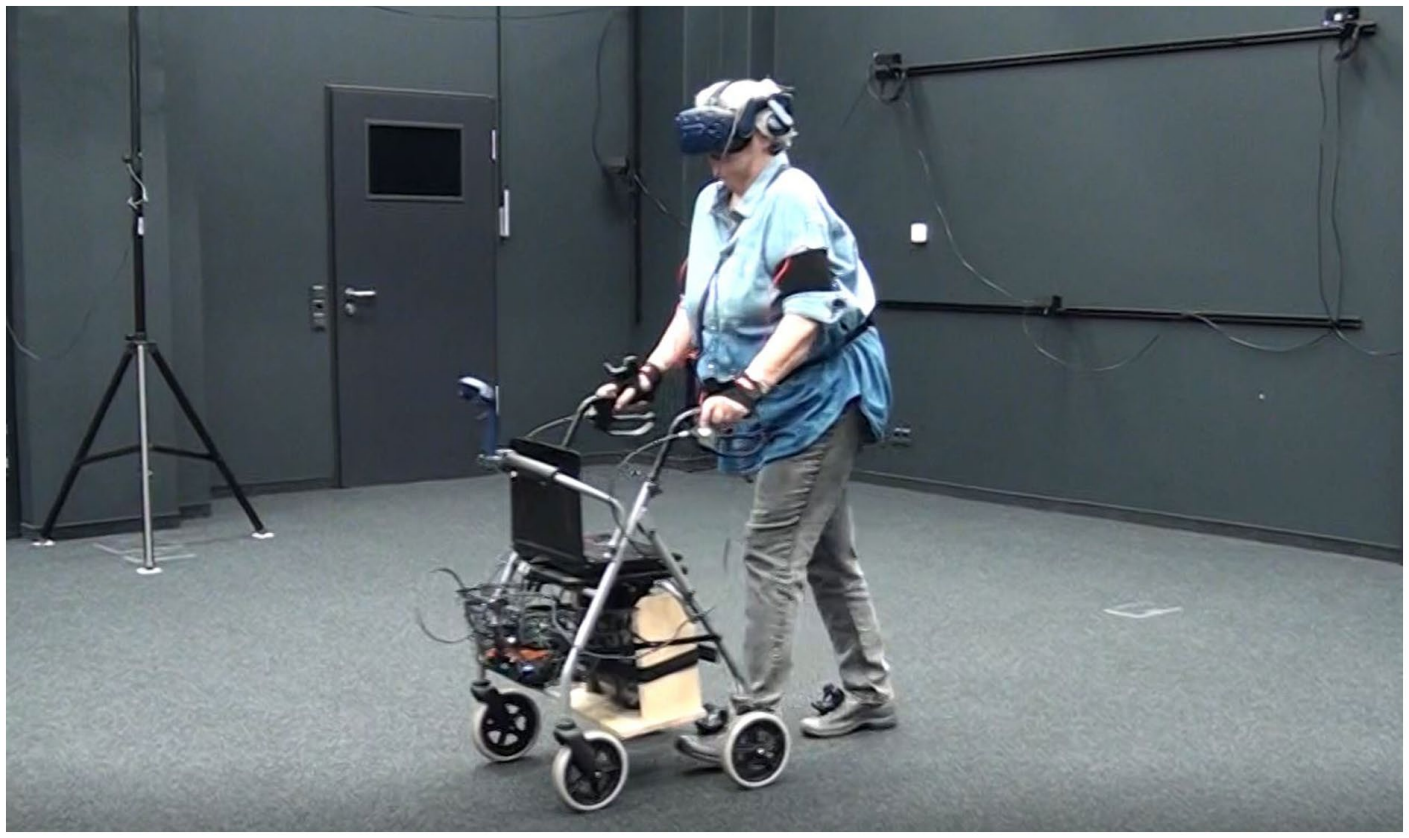
A participant wearing the HTC VIVE and using the walking frame during the VR experiment.

The prototype of the assistance system is shown in Figure 1. In two experiments, the assistance system was evaluated regarding its efficiency to increase safety during road crossing as well as with regard to the subjective workload and acceptance of the system. The first experiment was conducted in a virtual reality (VR) environment, and the second one was a field test.

## VR evaluation study

For a long time, pedestrian simulation has been mainly video-based and offered no or only short walking options. Since VR technology is evolving, more sophisticated pedestrian simulators have been developed, using head-mounted VR technologies. These simulation environments have a higher coupling of perception and action and allow for real walking. For a review see Feldstein et al. (2018).

The advantage of highly immersive VR experiments compared to video-based simulation environments is that they are much more realistic and, thus, provide results closer to real-life behavior. VR experiments allow for exposure of participants to traffic, without putting them in actual danger. Furthermore, they are more controlled than field tests. However, it has been shown that even with high fidelity simulation, participants still do not exactly behave as in real-world experiments (Feldstein et al., 2016). That is why we decided to combine both approaches, one controlled VR setting with actual traffic and one field test in a very quiet zone with little traffic.

The aim of the VR experiment was to investigate the behavior of older participants while road crossing with and without the assistance system. They walked up and down a 10 m long track and, during the walking, were presented with two different street scenarios. For their own safety, they were equipped with a walking frame during the whole experiment. The experiment was split into two parts, one where the assistance system was activated, and another one with the system turned off.

In both experiments, frequency of turning the head to the left and the right, stopping in front of the street, i.e., approaching time, workload measures, and acceptance questions served as dependent variables. It was expected that older pedestrians would stop more often and move their heads more often when using the system than when not using it. The workload measure was done to check whether the assistance system would increase the workload as an unintended side effect. No explicit hypotheses regarding acceptance questions were made. They rather served to learn more about participants’ attitudes towards the assistance system.

### Materials and methods

“Ethik-Kommission des Instituts für Psychologie und Arbeitswissenschaft (IPA) der TU Berlin” approved the study under the name: “VR-Studie zur Wirksamkeit eines vibro-taktilen Assistenzsystems für die Straßenquerung“(serial numbers WI_06_20180817). All procedures were performed in accordance with the Declaration of Helsinki, in compliance with relevant laws and institutional guidelines. Written informed consent was obtained from each participant and privacy rights were observed.

#### Participants

Twenty-three older subjects between the age of 65 and 83 (*M* = 73.3; *SD* = 5.6) were included in the analysis of this study. Ten of them were male and 13 were female. They all walked on foot on a regular basis. Participants were recruited *via* a participant tool of the research group fans. For participation, they received a compensation of 12€ per hour.

#### Research environment

The experiment took place in the “Berlin Mobile Brain/Body Imaging Lab” (BeMoBIL) of the Dep. of Biological Psychology and Neuroergonomics, Technische Universität Berlin. Participants wore HTC VIVE VR glasses and were additionally equipped with five trackers (feet, hands, and belly). The trackers and the glasses were tracked by a room-wide installed camera system. Figure 1 presents a participant with HTC VIVE during the experiment.

**Figure 2:**
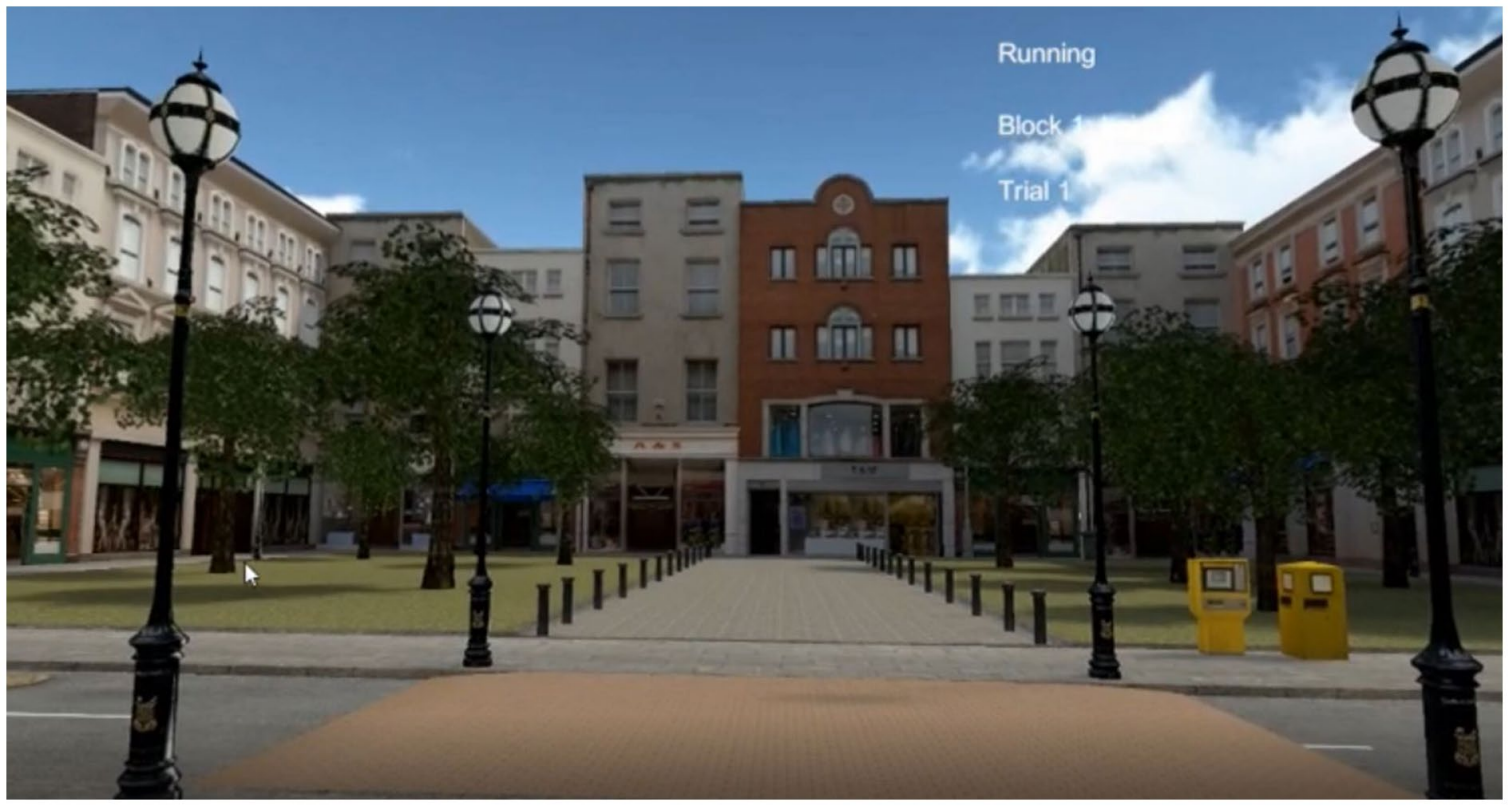
Street scene 1 of the VR experiment.

**Figure 3:**
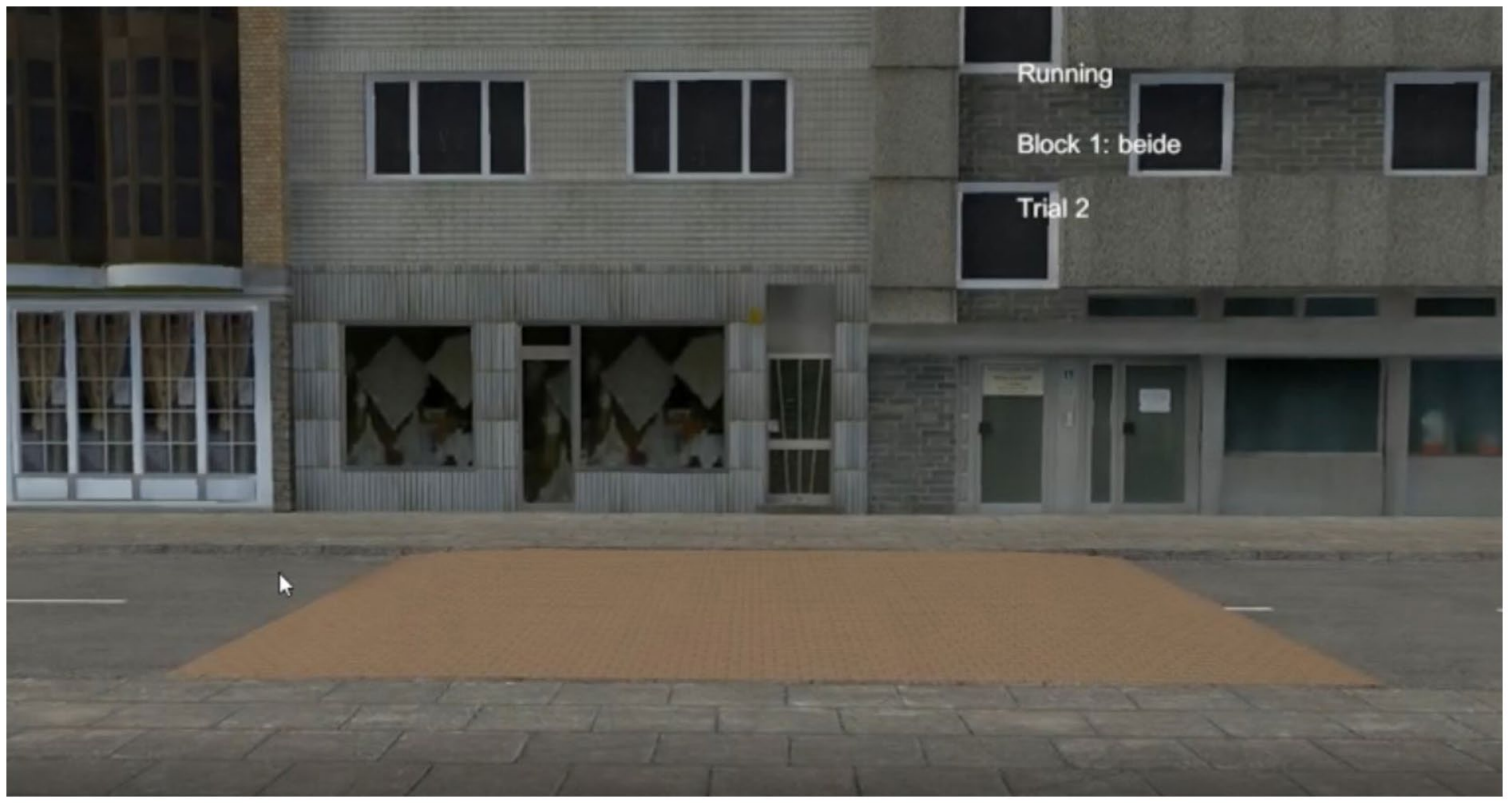
Street scene 2 of the VR experiment.

The scenarios were programmed with Unity. The VR scenes covered a 10 m × 5 m corridor. Two street scenarios had been developed for the experiment. Both showed urban environments. Pictures of each scene are presented in Figures 2, 3. For reasons of safety and logistics, it was decided not to use a height difference between the street and the footpath. Instead, the crossing consisted of a so-called “drop kerb,” which in Germany is often realized by raising the street instead of lowering the kerbstone. That allowed participants to walk on even ground through the whole scene, with a consistent view in the VR.

The whole walking distance inside the VR environment was 10 m, of which 7 m were in the street scene and 1.5 m to turn around at each end. Participants started 3.5 m before the curb stone. Vibration feedback was triggered when the subject was 2.25 m away from the kerbstone. This distance was chosen for practical reasons to assure enough time to check for cars. When entering the street, participants walked 3.5 m until the scene stopped automatically (0.5 m before the end of the virtual street).

In half of the scenes, cars were crossing. They crossed before and/or after the vibration feedback. Cars drove with a velocity of 28 km per hour. Cars appeared in a pseudorandom order. The time the cars started was varied to make prediction impossible for participants. Cars were triggered by the distance of the subject to the street. This distance varied between 5.5 m, 4 m, and 3 m before the vibration feedback, and 2 m, 1.5 m, and 1 m after the vibration feedback was given. The blocks consisted of 18 trials each, with nine trails containing cars. As it was a double lane road, cars could come from both directions. The number of cars from the left and right was counterbalanced.

When a new scene started, participants could decide when to start walking. When participants arrived at the other side of the road (i.e., 0.5 m before the end of the road), the scene ended, participants went into a grey room, where they received text-based information in addition to a symbol that indicated to turn around. They were then instructed to place their feet at a marked position on the floor. When they were in the right spot, the next scene started. The two different street environments were alternating.

The subject’s body was represented by either a male or female avatar to improve immersion (Slater, 2009). The representation of the walking frame, followed the hybrid prototyping approach (Exner et al., 2016). It was physically present and touched by the subjects, as well as also equipped with a tracker and visually represented in the VR.

The original assistance system that detects the kerbstone was simulated in the VR experiment. Thus, unlike the real system the one used in the VR experiment was 100% reliable.

#### Procedure

At arrival, participants filled in the consent form and read the instructions. Afterwards, they conducted the MoCA (*Montreal Cognitive Assessment*, Nasreddine et al., 2005), an acuity test (*Landolt ring chart*), and a test regarding contrast sensitivity (*Pelli-Robson chart*), before answering a simulator sickness questionnaire. Then, participants read the VR instruction, and trackers were put on the hands, feet, and belly. The avatar was calibrated to the person’s height. When everything was prepared, participants had a 10- to 20-min training phase to familiarize themselves with the VR environment. Before and after the training phase, they answered the SSQ (*Simulator Sickness Questionnaire*, Kennedy et al., 1993). Afterwards, the assistance system was introduced and its functions were demonstrated. Participants were instructed to cross the streets as normally as possible, i.e., to take safe decisions, but not to be unnaturally cautious. They were informed that the assistance system was there to support road crossing. However, they did not receive any instruction on how to behave as a response to the vibration signal. It was not mentioned that the system should support stopping and checking for traffic. The actual experiment consisted of two blocks with 18 trials each. Both blocks contained nine trials with cars and nine trials without. The walking frame was used during the entire experiment for safety reasons, but one block was conducted with the assistance system switched on, and the other one with the system switched off. The order of blocks was counterbalanced. After each block, participants filled in the NASA TLX (*NASA Task Load Index*, Hart and Staveland, 1988). When the experimental blocks were over, they answered the acceptance questionnaire. Finally, participants received financial compensation and were thanked for their participation.

#### Dependent measures

Objective dependent measures were stopping (both feet on the floor with a max. length of 5 cm between feet, for a min. time of 2 s) frequency per block and head rotation frequency (straight, medium left, medium right, complete left, and complete right). Every orientation was defined as a window of 36° in the rotation field of 180° in front of the participant. NASA TLX served as a measure for subjective workload. Acceptance was assessed *via* the three questions that are listed in Table 1.

**Table 1:** Frequencies and percentages of answers to the three acceptance questions regarding the assistance system in the VR study.

Questions	Answers									
The assistance system increases traffic safety	Totally disagree		Rather disagree		Indifferent		Rather agree		Totally agree	
	1	4.3%	2	8.7%	4	17.4%	9	23.1%	7	30.4%
A lot of people would like the assistance system	Totally disagree		Rather disagree		Indifferent		Rather agree		Totally agree	
	0	0%	5	21.7%	5	21.7%	11	47.8%	2	8.7%
Would you buy such a system?	Most unlikely		Rather unlikely		Indifferent		Rather likely		Most likely	
	4	17.4%	2	8.7%	10	43.5%	3	13%	4	17.4%

### Results

Stopping frequency and workload were analyzed with 2 × 2 ANOVAs with repeated measures. Head rotation frequency was analyzed with a 2 × 2 × 5 ANOVA. Significance level alpha was set to 0.05. Values between 0.05 and 0.1 are classified as marginally significant. Acceptance was analyzed descriptively. Assumptions of sphericity were tested using the Mauchly test. In case of violation, Greenhouse–Geisser corrected values are reported.

#### Stopping frequency

**Figure 4:**
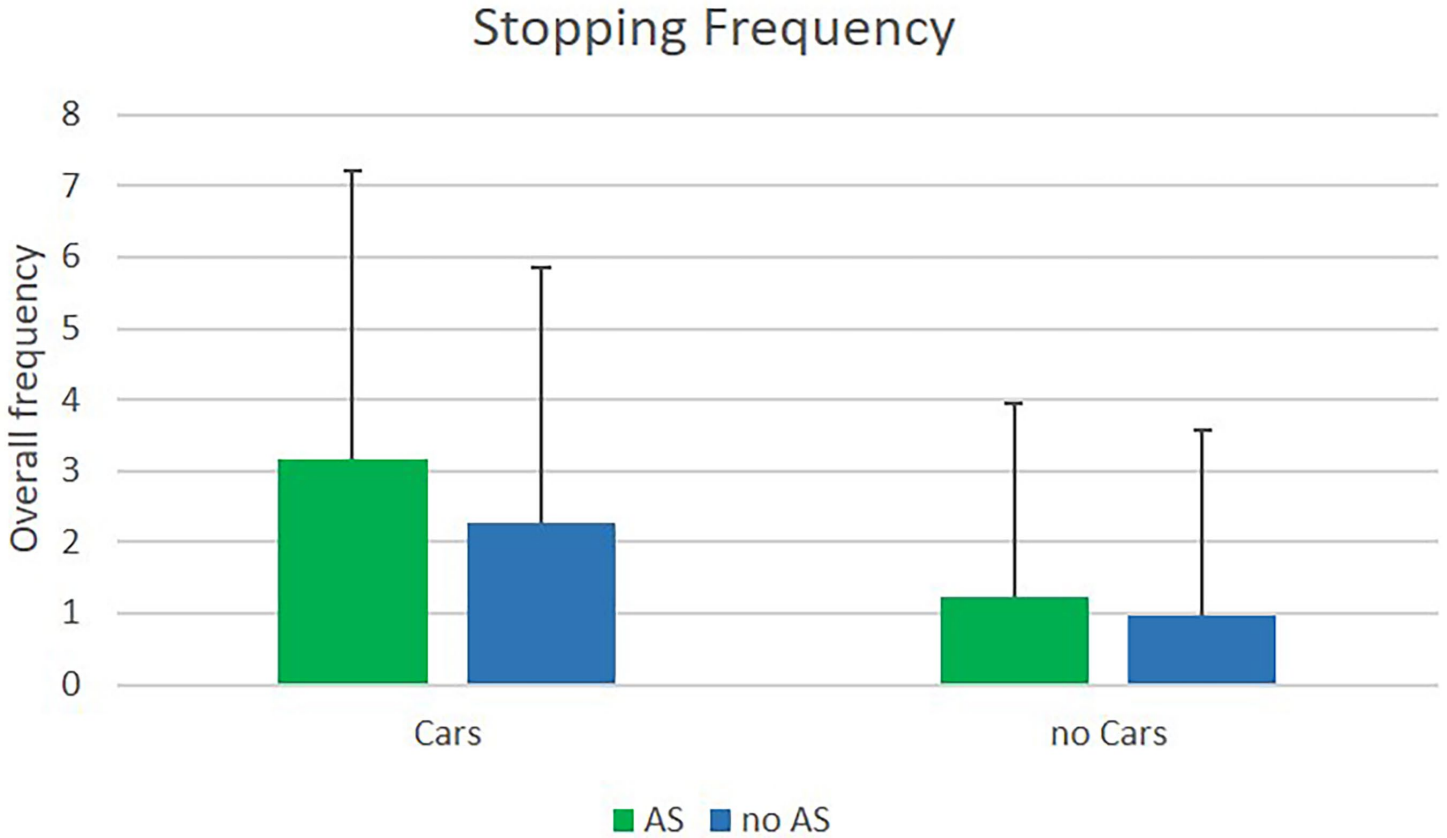
Means of stopping frequencies in the VR experiment with and without cars, with (AS) and without (nAS) the assistance system.

Stopping frequencies were analyzed using the sum of all stops for the respective number of trials (i.e., 18 trials with/without cars, and 18 trials with/without an assistance system). The main effect for cars revealed significance with a large effect size, *F*(1, 22) = 9.27; *p* = 0.006; *η^2^_p_* = 0.3. When cars were crossing, participants stopped with a higher frequency (*M* = 2.72 *SD* = 3.82) than without cars (*M* = 1.09; *SD* = 2.67), but the standard deviation was higher with cars than without. Analysis of the main effect of the assistance system revealed only a marginally significant result but had a large effect size, *F*(1, 22) = 3.53; *p* = 0.07; *η^2^_p_* = 0.14. Participants stopped more often with the assistance system (*M* = 2.2, *SD* = 3.38) than without it (*M* = 1.63, *SD* = 3.1), and the standard deviation was similar for both conditions. The interaction effect did not reveal significance. Results are presented in Figure 4.

#### Head rotation frequency

**Figure 5:**
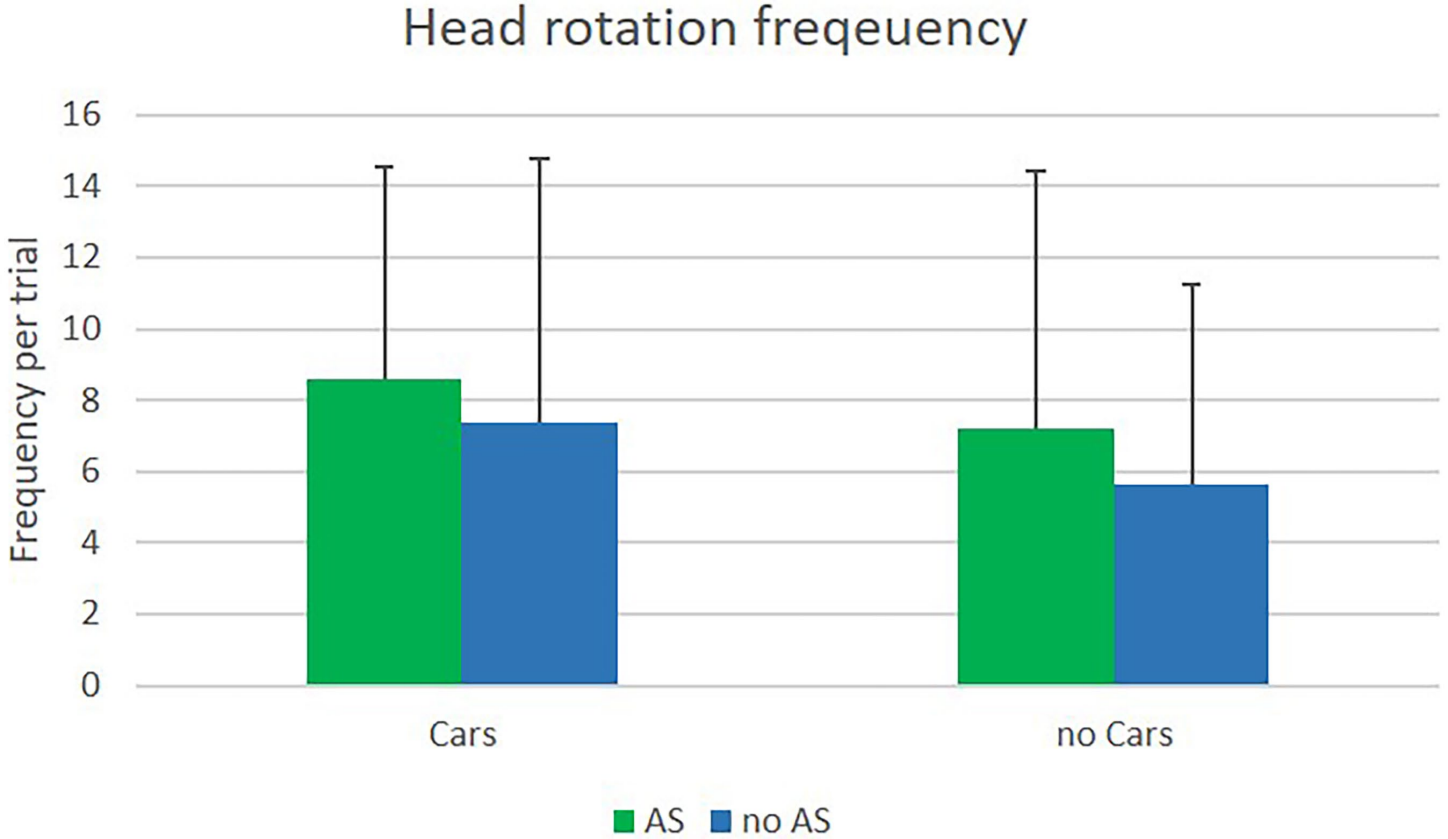
Means of head rotation frequencies in the VR experiment with and without cars, with (AS) and without (nAS) the assistance system.

**Figure 6:**
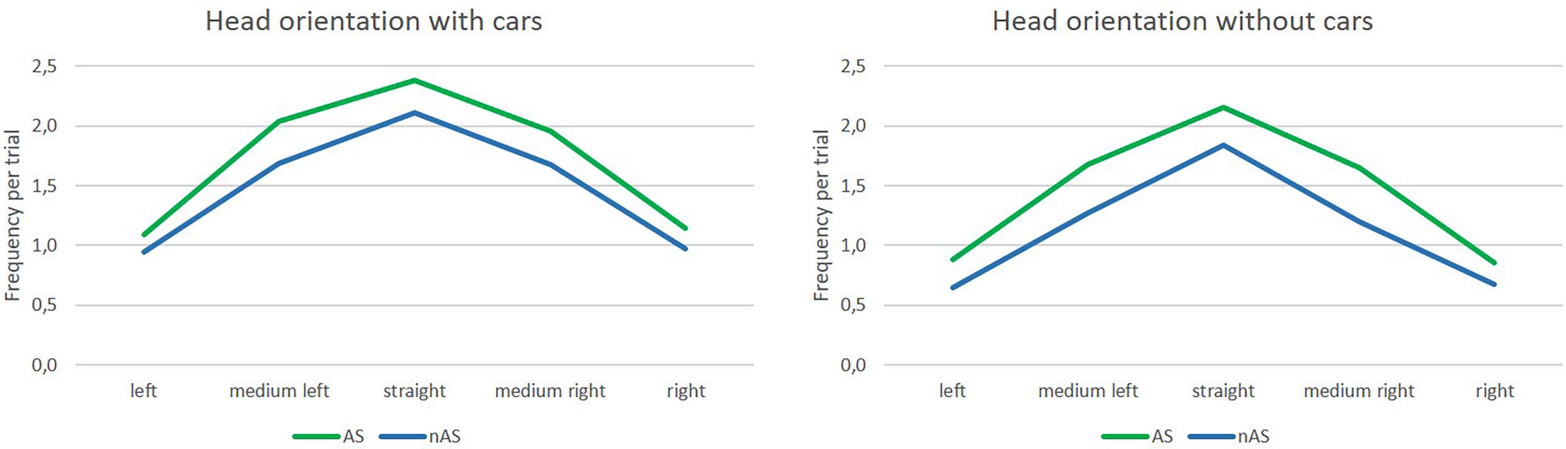
Means of frequencies of different orientations of head rotation in the VR experiment with and without cars, with (AS) and without (nAS) assistance system.

Head rotation frequencies were analyzed for the five orientations within single trials. The main effect of cars was significant and based on a large effect size, *F*(1, 22) = 7.68; *p* = 0.01; *η^2^_p_* = 0.26. Participants moved their heads more frequently when cars were passing (sum of all orientations: *M* = 8.0, *SD* = 6.1) than without cars (sum of all orientations: *M* = 6.4, *SD* = 4.16), but the standard deviation was higher with cars. The main effect of the assistance system revealed significance and the effect size was large, *F*(1, 22) = 5.72; *p* = 0.03; *η^2^_p_* = 0.21. The frequency of head movement was higher with the assistance system (sum of all orientations: *M* = 7.92, *SD* = 5.14) than without the system (sum of all orientations: *M* = 6.51, *SD* = 5.07), and the standard deviation was similar for both conditions. Results are presented in Figure 5. The main effect for orientation was also significant with a very large effect size, *F*(1.76, 38.81) = 44.78; *p* < 0.001; *η^2^_p_* = 0.67. The highest frequency was found for the straight head orientation (*M* = 2.12, *SD* = 1.16), followed by the medium right (*M* = 1.62, *SD* = 1.17) and the medium left orientation (*M* = 1.67, *SD* = 1.38), and the lowest frequencies were found for complete right (*M* = 0.91, *SD* = 0.66) and complete left orientations (*M* = 0.89, *SD* = 0.74). The interaction between the assistance system and orientation was marginally significant, with a medium effect size *F*(2.39,52.52) = 2.82; *p* = 0.06; *η^2^_p_* = 0.11. The other interactions were not significant. Results are presented in Figure 6.

#### Workload

The overall workload did not differ significantly. It was perceived as low (on a scale of 0–100) in both conditions, with the assistance system (*M* = 10.17; *SD* = 10.1), and without it (*M* = 11.96; *SD* = 10.96). The workload on the single scales was not significantly different as well.

#### Acceptance

Participants were asked three questions regarding acceptance of the assistance system, which are analyzed descriptively. When being asked whether the assistance system “increases traffic safety,” 70% of the participants indicated that this was rather true or totally true. Moreover, 57% stated that it was rather true or totally true that “a lot of people would like the assistance system.” However, when asked how likely they would be to “buy such an assistance system,” only 31% thought that was rather or most likely. Response frequencies are presented in Table 1.

### Discussion

In this evaluation study, the prototype of an assistance system has been evaluated with regard to its capacity to change users’ behavior towards safety. The aim of the system was to make users stop more frequently and to check for traffic more often. Results indicate that the system had a positive effect on both.

Participants stopped more often when the assistance system was activated than when it was switched off. However, the total numbers for stopping with the assistance system are still very low (medium of 2.2 in 18 trials).

Participants’ head rotation was used to measure their frequency of looking for traffic. The assistance system increased rotation frequency, independent of orientation. When the system was switched on, participants looked for traffic more often. This behavior was shown without explicit instructions on how to behave in response to the vibration signals.

In addition, it was shown that the approaching cars were an external trigger for stopping as well as for head rotation, which is plausible. Interestingly there was no interaction between the assistance system and crossing cars. That means, the system did not only improve behaviors that were already in place but did trigger the safer behavior also in trials without crossing cars.

#### Limitations

The floor of the laboratory environment was free from any steps and other potential obstacles in both the external and the VR vision. Thus, the main reason for engaging in parallel visual tasks, checking the ground to prevent falling, was not really an issue in this setting. Thus, it is possible that frequency of head rotation would be lower in an environment that requests more visual checking of the floor, as was the case in the field test.

From a theoretical point of view, the experiment could only show an increase in stopping and an increase in head rotation behavior. With the current setup, it was not possible to combine these two measures to understand whether they are related. Further studies should investigate whether the increase in head rotation is higher during the time the participants stop in order to allow for a valid interpretation regarding the reduction of dual-task activities.

## Field test

Based on the promising results from the VR study, the next step was to validate its results in a real street environment, i.e., to evaluate the prototype of an assistance system in the field. The behavior of pedestrians differs dependent on the type of crossing, especially with regard to marked crossings (e.g., zebra, sunken curb) versus other crossings (e.g., Schüller et al., 2020). There is considerable dissent in the literature regarding the advantage of marked crossings. On the one hand, crossing where there is no official crossing is unexpected for drivers and, thus, adds the risk of drivers overlooking pedestrians. On the other hand, pedestrians behave less carefully at marked crossings, because they expect drivers to stop, which is not always the case. To investigate whether participants’ behavior differed at marked versus unmarked crossings and, more importantly, to investigate whether this could have an impact on the use of the assistance system, about half of the crossings in the experiment were marked, and the other half were unmarked.

The current experiment does not focus on road crossing behavior, but rather on the use of the assistance system. When being in a completely new situation, such as a VR environment, the use of a walking frame that is normally not used, may not have the same (potentially distracting) impact as it can have in the real-world. To reduce the impact of the walking frame, participants were using it in both conditions, with and without the assistance system, as was also done in the VR study.

While there are, of course, already a lot of differences between an experiment in VR and the field, in this case there was another very important difference regarding the assistance system. The aim of these two experiments was not to compare behavior in the VR and the real environment but to evaluate the prototype of the assistance system with the best combination of internal and external validity. While the assistance system in the VR study was perfectly reliable because its signals were triggered by the simulation software, the assistance system used in the field test was a real functioning prototype. Thus, there are a lot of possibilities for errors made by the system (missing the curb stone as well as generating false alarms), that can have an impact on the behavior of the participants.

Results regarding the comparison of VR and pedestrians in real environments are mixed. Schwebel et al. (2008) suggest that decisions in VR and real street environments were highly correlated. However, Feldstein (2019) argues that the absolute numbers can still differ significantly, even being highly correlated. Feldstein and Dyszak (2020) found participants in the VR to take riskier crossing decisions compared to the real-world.

Based on the previous studies that found similar but safer behavior in real environments (Schwebel et al., 2008; Feldstein, 2019; Feldstein and Dyszak, 2020) compared to VR, it was expected to find the same behavioral patterns. The workload was assessed to make sure that the assistance system did not impose additional workload on the users. Three questions regarding the acceptance of the system were asked at the end of the experiment to learn more about users’ needs and potential drawbacks of using an assistance system.

### Materials and methods

“Ethik-Kommission des Instituts für Psychologie und Arbeitswissenschaft (IPA) der TU Berlin” approved the study under the name: “Studie zur Nutzung eines Fußgängerassistenzsystems im Straßenverkehr“” (serial numbers BRE_02_201808803). All procedures were performed in accordance with the Declaration of Helsinki, in compliance with relevant laws and institutional guidelines. Written informed consent was obtained from each participant and privacy rights were observed.

#### Participants

Twenty-six older subjects between the age of 65 and 85 (*M* = 73.15; *SD* = 5.38) were included in the analysis of this study. Thirteen of them were male, 12 were female, and one preferred not to say. They all walked on foot on a regular basis. Participants were recruited *via* a participant tool of the research group fans. For their participation, they received compensation of 12€ per hour.

#### Procedure

Participants started the experiment in a laboratory room at the university. Upon arrival, participants filled in the consent form and read the instructions. Afterwards, they conducted the MoCA (*Montreal Cognitive Assessment*, Nasreddine et al., 2005), and an acuity test (*Landolt ring chart*). The walking frame was used during the entire experiment to keep the situation comparable with regard to walking speed, etc. One way was conducted with the assistance system switched on, and the other one with the system switched off. The order of system use was counterbalanced. After all the tests had been conducted, participants were brought outside. A helmet equipped with a GoPro camera and vibration cuffs were placed on the participants. The functioning of the assistance system was demonstrated, and, subsequently, participants had time to familiarize themselves with the walking frame and the assistance system. When they were ready, the experiment started. Participants started at the university and walked around the surrounding streets. In this area, there is rather low traffic. The route they had to follow was marked with chalk on the floor. They walked alone but knew that the experimenters were nearby to help them in case they needed it. When they arrived, they sat down on a bench with the experimenters and filled in the SEA scale. After a break, they went the same way back. When participants came back to the starting point, they answered the SEA scale again as well as the acceptance questions. Finally, participants received financial compensation and were thanked for their participation.

#### The assistance system

**Figure 7:**
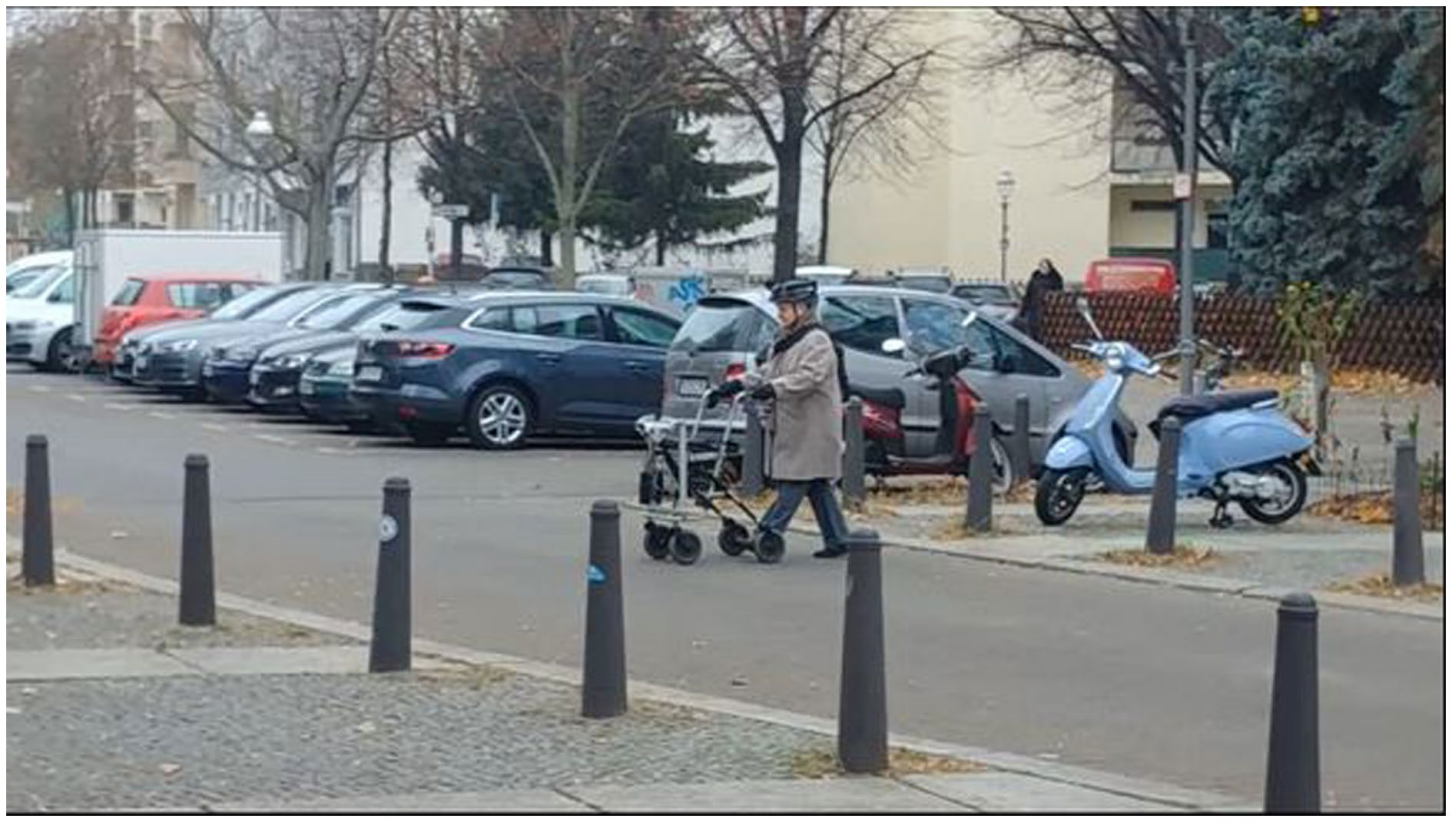
Participant in the field study using the walker with the assistance system at a marked crossing.

**Figure 8:**
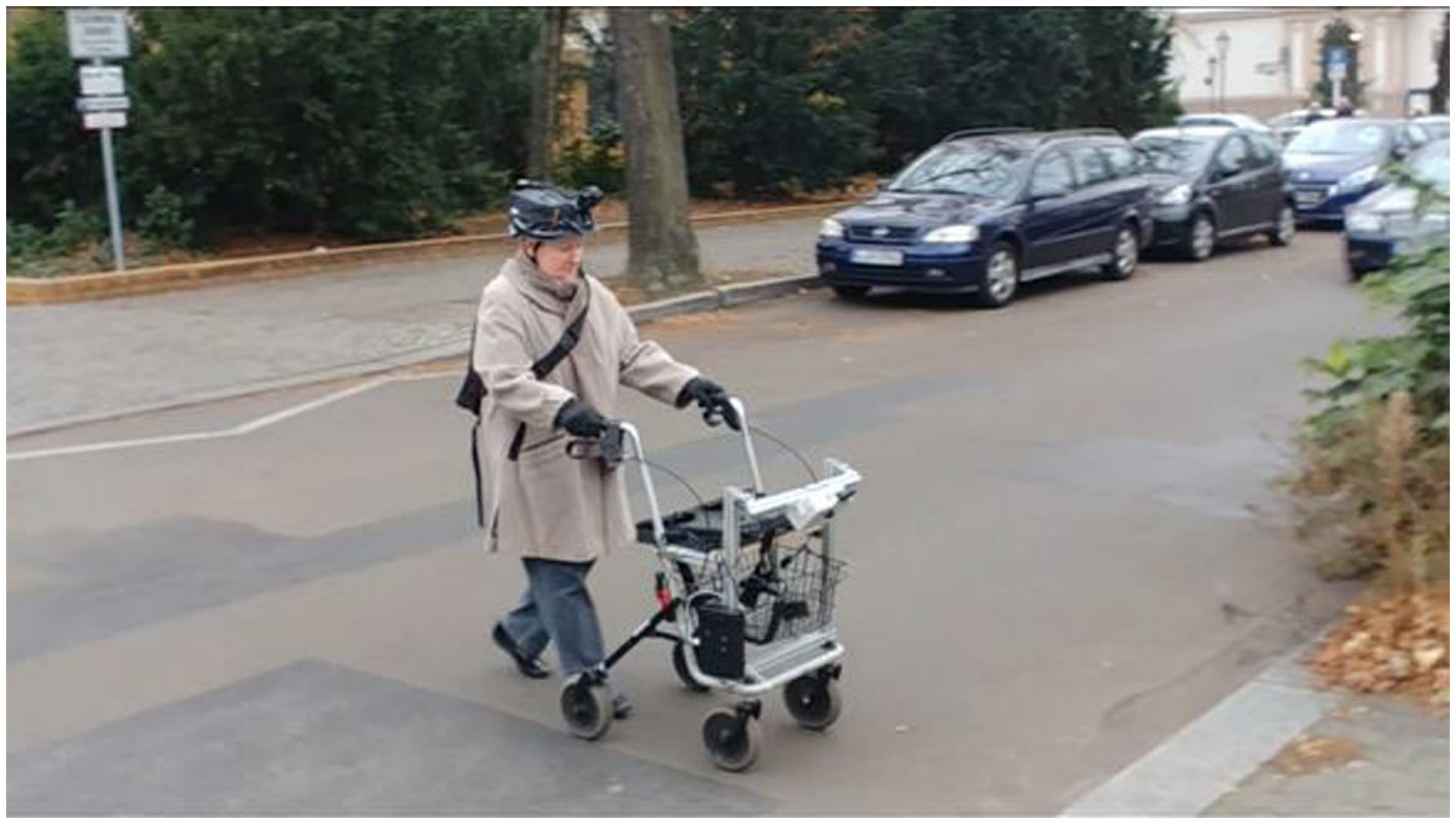
Participant in the field study using the walker with the assistance system at an unmarked crossing.

The prototype consists of two sensors, one webcam and one infrared sensor, a laptop, and an Arduino, as well as two vibration cuffs. Sensors and laptop are mounted to a walking frame, the Arduino was placed in a backpack, carried by the participants, and vibration cuffs were placed on the upper arms of the users. The system needs about 1 s (i.e., 15 frames) to analyse the surroundings and to decide whether to generate an alarm or not. The system is programmed to detect the kerbstone within a predefined window of 2 m +/− 1 m. The actual distance depends on the approaching angel of the person and can thus vary between trials. If the system detects a kerbstone it generates vibration signals at the cuffs *via* the Arduino. The signal duration was 500 ms. Figures 7, 8 show a participant using the walker with the assistance system. After an alarm was generated, the system did not generate another alarm within a time window of 5 s. This was done to avoid continuous alarms when participants had to wait at the street. The system had a reliability of over 99% with the test data (Qureshi and Wizcorek, 2019). However, in the real street environment, a lot of objects were present, which had not been part of the training data (trees, people, etc.) that caused the system to generate false alarms. Since the system had not yet been trained for this data and because it is not possible to calculate a false alarm rate (the underlying number of events is unknown), correct rejections (true negatives), and false alarms (false positives) are not analyzed. Instead, the hits (true positives) and the misses (false negatives) of the current study are used to calculate the hit-rate of the system, which can be compared to the validation data from the laboratory (Qureshi and Wizcorek, 2019). A more detailed analysis of the functional part of the system in this study can be found elsewhere (Qureshi, submitted).^2^

#### The route

The route was 0.5 km long and included 13 crossings, of which seven were marked and six were unmarked. Examples of crossings can be seen in Figures 7 (marked), Figure 8 (unmarked). The route consisted of normal crossings in a normal street environment but was in a quiet area with low traffic density. In the streets with the unmarked crossings, there existed no marked crossings nearby, so all the pedestrians in this area were using these crossings on a frequent basis. Unmarked crossings were chosen in spots where people normally cross and it was made sure that the spots were comparatively safe (free view, no junctions or merging traffic, etc.).

#### Dependent measures

Dependent measures were the following: Objective measures were head rotation frequency (left vs. right) per trial and approach duration when walking towards the street. Head rotation was measured *via* IMU (Inertial Measurement Unit). As it turned out after the experiment that it was impossible to measure stopping frequency *via* IMU, due to the near nonappearance of this behavior, it was decided to use the approaching time as an objective behavioral measure instead. This was assessed *via* a GoPro camera. The workload was assessed using the SEA scale (Eilers et al., 1986). The scale consists of a vertical scale from 0 to 220 with verbal anchors. The SEA scale was chosen instead of the NASA TLX because it can be filled in faster. As the experiment took place between November and December, it was aimed to keep the time participants had to sit in the cold to answer questionnaires as short as possible. Acceptance was assessed *via* three questions that are listed in Table 2.

**Table 2:** Frequencies and percentages of answers to the three acceptance questions regarding the assistance system in the field study.

Questions	Answers									
How did you perceive the assistance system during road crossing?	helpful		Rather helpful		irrelevant		Rather disturbing		disturbing	
	1	3.7%	7	25.9%	15	55.6%	3	11.1%	0	0%
Would you recommend such a system to an older person in need of support with road crossing	yes		Rather yes		indifferent		Rather no		no	
	13	48.1%	3	11.1%	5	18.5%	3	11.1%	2	7.4%
Would you buy such a system?	yes		Rather yes		indifferent		Rather no		no	
	2	7.4%	3	11.1%	2	7.4%	8	29.6%	11	40.7%

### Results

Head rotation frequency, approaching time, and workload have been analyzed with ANOVAs for repeated measures. Significance level alpha was set to 0.05. Values between 0.05 and 0.1 are classified as marginally significant.

#### Hit-rate of The assistance system

According to the signal detection theory (*cf.* Swets, 1964), the hit-rate of a system indicates how well the system detects the defined targets. The hit-rate of the current experiment ranged from 0.769 to 1 with *M* = 0.922, and *SD* = 0.068. This means that the system failed to generate an alarm in 8% of the cases in which a person approached a street. The actual hit-rate was significantly lower than the hit-rate of 0.988 that was reached in the laboratory (Qureshi and Wizcorek, 2019), *t*(51) = −6.944; *p* < 0.001.

#### Head rotation frequency

**Figure 9:**
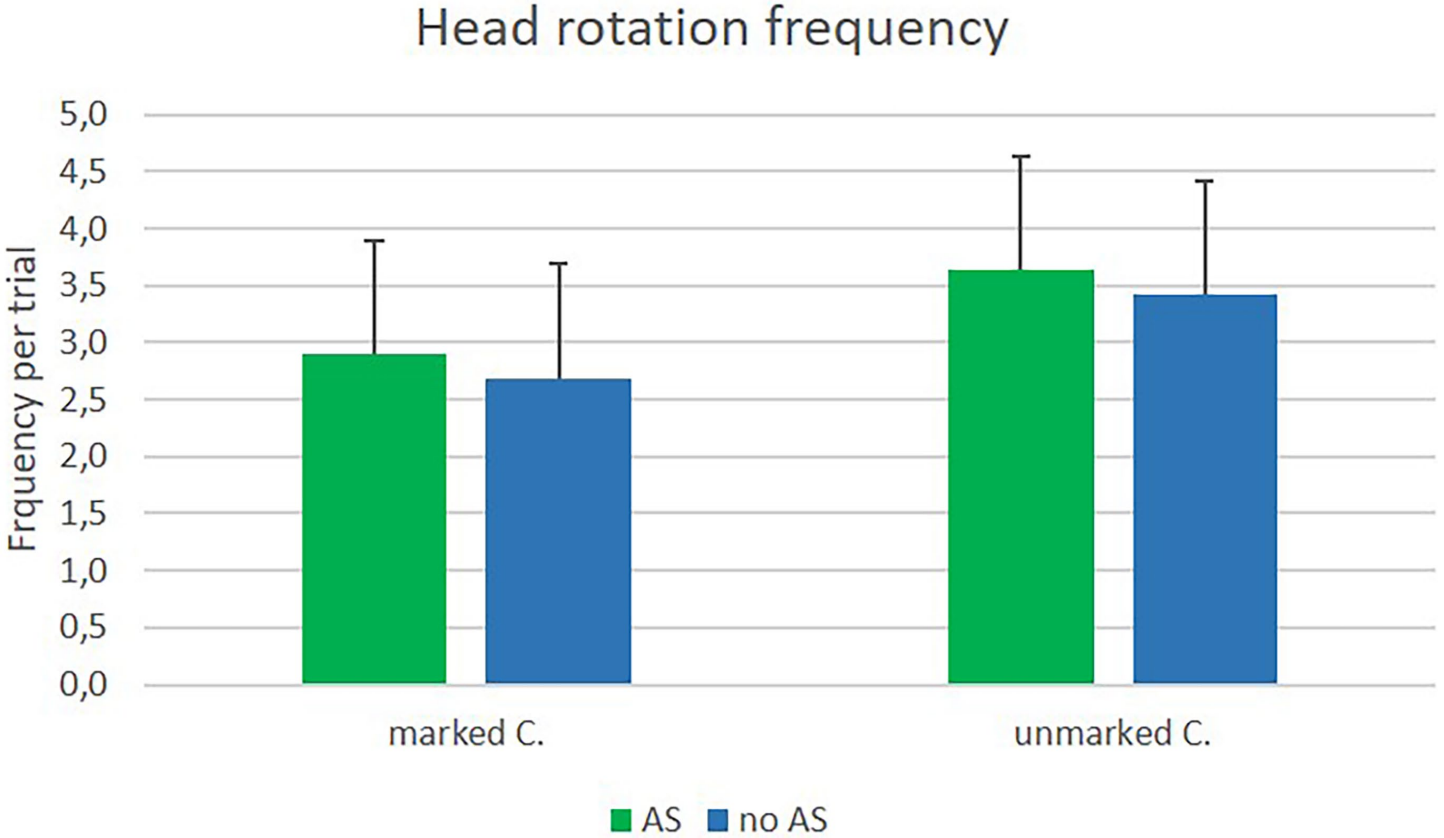
Means of head rotation frequencies in the field experiment at marked and unmarked crossings with (AS) and without (nAS) assistance system.

**Figure 10:**
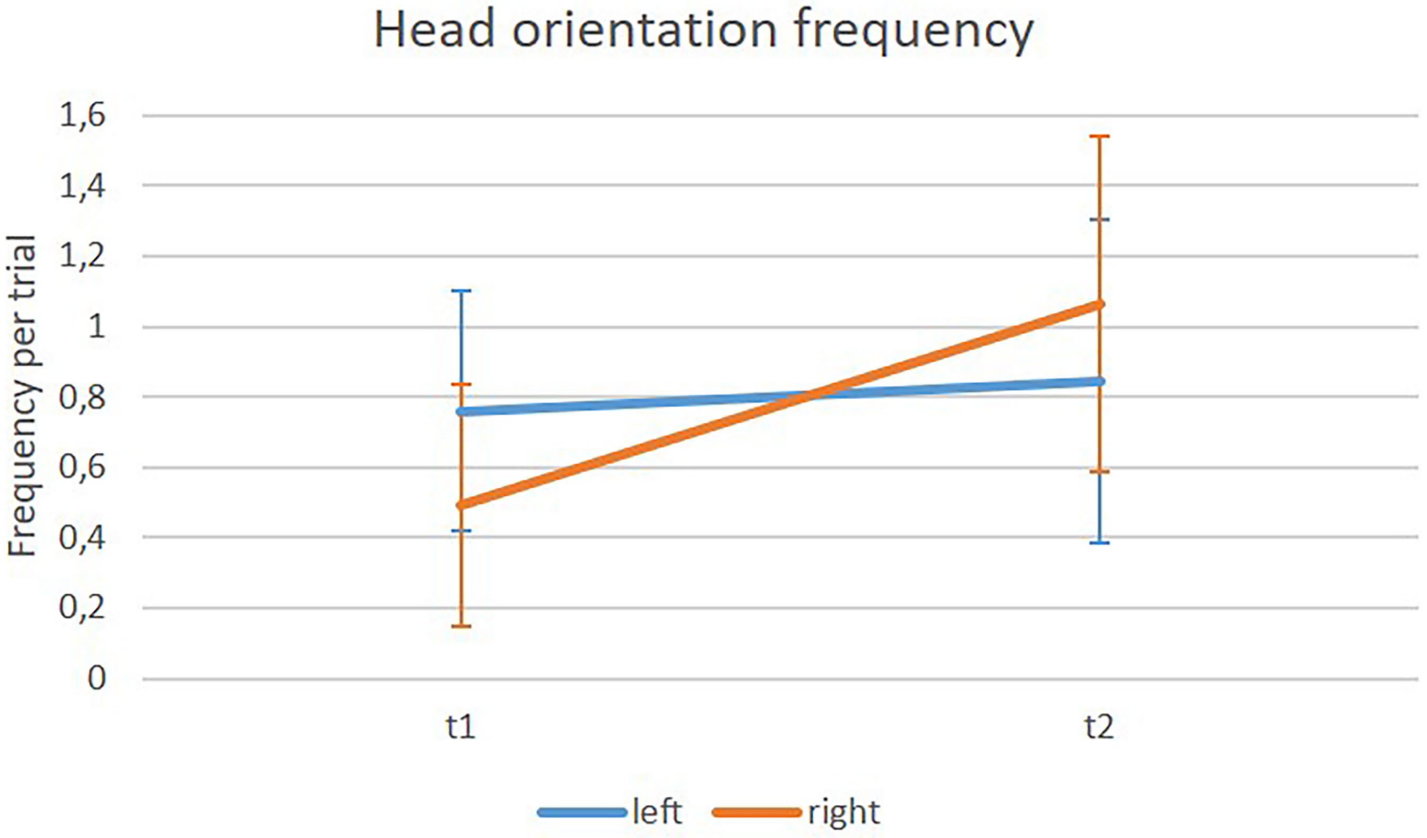
Means of frequencies of different orientations of head rotation in the field experiment on the footpath (t1) and on the street (t2).

Rotation frequency was analyzed per trial. The main effect of assistance system was marginal significant, *F*(1, 25) = 3.60; *p* = 0.07; *η^2^_p_* = 0.13. Participants moved their heads more often when using the assistance system (*M* = 0.82, *SD* = 0.4) than without the assistance system (*M* = 0.76, *SD* = 0.41). The main effects for type of crossing, *F*(1, 25) = 35.41; *p* < 0.001; *η^2^_p_* = 0.59, and for position, *F*(1, 25) = 15.24; *p* = 0.001; *η^2^_p_* = 0.38, revealed significance, but were further qualified by two interaction effects; namely the significant interaction between type of crossing x position, *F*(1, 25) = 105.18; *p* < 0.001; *η^2^_p_* = 0.81, and the significant interaction between position x head orientation, *F*(1, 25) = 54.52; *p* < 0.001; *η^2^_p_* = 0.69. The first implies that participants showed a different pattern of head movement depending on the type of crossing. When at a marked crossing, they moved their heads more often when still being on the footpath and less often while on the street (marked crossing/footpath: *M* = 0.77, *SD* = 0.37; marked crossing/street: *M* = 0.63, *SD* = 0.4). The opposite was found for unmarked crossings. Participants moved their heads less often when still on the footpath and more frequently while already on the street (unmarked crossing/footpath: *M* = 0.49, *SD* = 0.32; unmarked crossing/street: *M* = 0.1.28, *SD* = 0.53). The second interaction describes that participants looked more often to the left than to the right side, when still being on the footpath (footpath/left orientation: *M* = 0.76, *SD* = 0.34; footpath/right orientation: *M* = 0.49, *SD* = 0.34), while doing the opposite while on the street; looking more often to the right than to the left side (street/left orientation: *M* = 0.85, *SD* = 0.46; street/right orientation: *M* = 1.06, *SD* = 0.48). The main effect of orientation was not significant. Results can be seen in Figures 9, 10.

#### Approaching time

**Figure 11:**
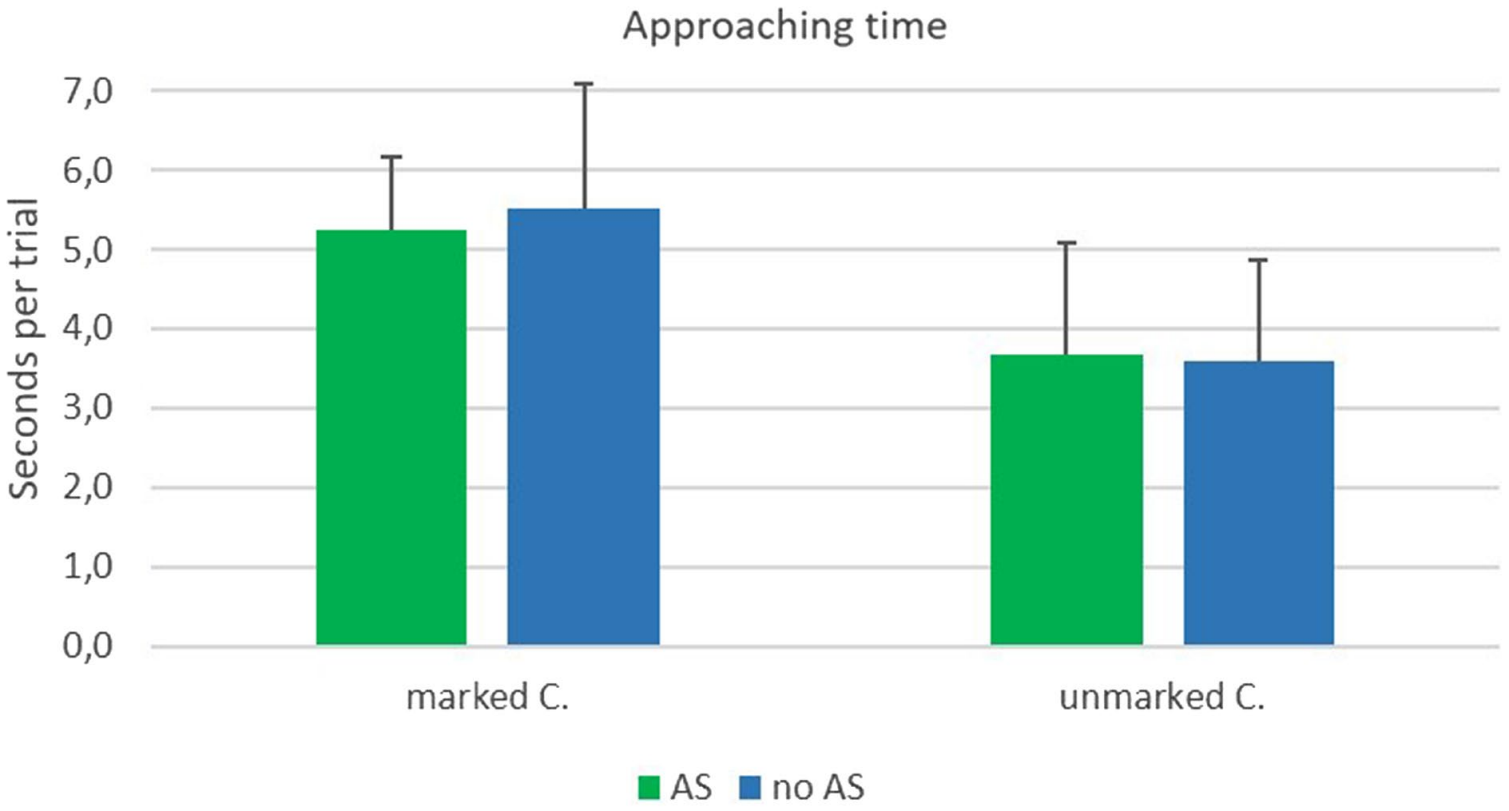
Means of approaching time in the field experiment at marked and unmarked crossings with (AS) and without (nAS) assistance system.

The main effect of type of crossing revealed significance, *F*(1, 25) = 117.38; *p* < 0.001; *η^2^_p_* = 0.82. The approaching time was longer at marked crossings (*M* = 5.39 s, *SD* = 1.23 s) compared to unmarked crossings (*M* = 3.64, *SD* = 1.34). The main effect of assistance system and the interaction effect did not reveal significance. Results can be seen in Figure 11.

#### Workload

The workload assessed with the SEA scale did not differ significantly. It was perceived as low (on a scale of 0–220) in both conditions, with the assistance system (*M* = 25.0; *SD* = 23.93) and without it (*M* = 25.87; *SD* = 22.85).

#### Acceptance

Participants were asked three questions regarding acceptance of the assistance system, which are analyzed descriptively. When being asked, “how did you perceive the assistance system during the road crossing?,” only 30% answered helpful or rather helpful, while more than 50% considered it irrelevant. Consistently, when being asked how likely they would be to “buy such an assistance system for themselves,” 70% stated they would not or rather not. However, when being asked whether they would “recommend such a system to an older person with problems in road crossing,” almost 60% said yes or rather yes. Response frequencies are presented in Table 2.

### Discussion

Results regarding head rotation offer interesting insight into the crossing strategies of older pedestrians. In line with other research (*cf.* Schüller et al., 2020), a different behavioral pattern for marked and unmarked crossings was found. Participants in the current study moved their heads less often to check for traffic while at an unmarked crossing. Moreover, when crossing marked crossings, they checked for traffic more often when still being on the footpath before entering the street and less often when already on the street. The opposite was found for unmarked crossings. Participants did check less often before entering the street and more often when already being on the street. In addition, they looked to the left more often while still on the footpath and more often to the right, while on the street.

Of course, there may be systematic differences between marked and unmarked crossings that support this behavior apart from the type of crossing itself (the size of the road, the visibility, etc.). Thus, interpretation of different behavioral patterns at marked and unmarked crossings is difficult. However, the tendency to look more to the left on the footpath and more to the right on the street is independent of the type of crossing. This behavior may seem logical on a double lane road. However, it incorporates the risk of neglecting the right side until being physically on the street. A behavior that has been observed before for older pedestrians (*cf.* Dunbar et al., 2004). It is due to the age-related limitation of working memory, making it more difficult for older pedestrians to integrate information from the two orientations. When streets are bigger, islands are an important instrument to improve road crossing safety for older pedestrians. However, in small but still double lane streets, like the ones in the current experiment, it may be the only suitable strategy for older pedestrians. Unfortunately, the use of the assistance system cannot tackle this problem of limited workload. The only technological solution that could overcome this dangerous situation is the P2C-communication (pedestrian to car communication).

Results of the approaching time and type of crossing are in line with findings for head rotation. Participants behave differently at marked and unmarked crossings. A shorter approaching time at unmarked crossings corresponds to the finding of increased checking for traffic when being on the street. The assistance system, however, had no impact on the approaching time. It did not make people stop or even walk slower. In our opinion, the absence of any effect of the assistance system on approaching time may be the result of a very strong automation of the road crossing behavior that has been trained for years.

## General discussion

The aim of these two studies was to evaluate whether the prototype of the assistance system promotes safe behavior of older pedestrians. It was supposed to increase head rotation as the operationalization of looking for traffic and to increase stop frequencies to reduce multitasking requirements. The VR study offered the possibility to control the functioning of the assistance system and investigate the potential interaction of the assistance system with cars in a safe, but realistic environment. The field study served to test the functioning of the real prototype and to evaluate the impact of the assistance system on the behavior in a real-world environment as well as potential interactions with the type of crossing.

### Stopping frequency

Even though the effect of cars on stopping frequency was much higher than the effect of the assistance system, participants in the VR study stopped significantly more frequently when using the assistance system than without it. It is important to mention that this effect occurred without participants being instructed to do so and was also unrelated to the stopping of cars. This finding is encouraging because it can be interpreted as evidence for the intuitive design of the assistance system.

However, in the real-world environment, the assistance system did not approaching time speed significantly, much less did it increase stopping frequency (which could not be measured due to its rare occurrence).

In this measure, we found the only difference between the two evaluation studies. In our interpretation, the behavior shown in the VR resembles the normative road crossing behavior, including stopping at streets. The VR environment triggers behavior close to normal, but at the same time allows the participants to reflect and adapt their behavior. In the field study, however, it was not possible to interrupt the routine of checking for traffic while walking through the use of the prototype. It has to be noted, again, that people were not instructed to stop in response to the vibration signal. Thus, it is possible that the desired behavior could be achieved with the help of instruction and training.

### Head rotation

In contrast to the stopping frequency, i.e., approaching time, that could not be manipulated by the assistances system in the real-world environment, participants did significantly increase their head rotation in both experiments. The effect was stronger in the VR experiment (*η^2^_p_* = 0.21) compared to the field test (*η^2^_p_* = 0.13), where it was only marginally significant. One possible reason could be the flat and even ground in the VR experiment not requiring further visual attention unlike the real street environment. In both experiments, external factors such as passing cars and type of crossing did also increase the frequency of head rotation. However, in both cases no interaction with the effect of assistance system was found. Furthermore, the increase of head rotation frequency took place in both settings without instructing participants to do so. This finding is a major success in the development of the assistance system, and another strong evidence for the intuitive design of the prototype. However, as there are a lot of potential visual distractions in the real-world, it will be necessary to instruct and to train the users in order to achieve a long lasting behavioral change.

### Workload

It is very important to remember that new technical systems incorporate the risk of unintended negative side effects. One common problem is them imposing additional workload. Thus, it is a positive finding that the prototype of the assistance system did not increase workload in the VR or in the field study. In both experiments, the workload was experienced as low. That reflects the quotidian nature of the task.

### Acceptance

In line with the workload results, only 11% of participants in the field study reported that the system interfered with their task of road crossing, and more than half of the participants in the VR thought the system increased traffic safety. However, only 30% of participants in the field test felt the system would support them. Consistently, the likelihood of buying such a system for themselves was low in both studies (VR: 26%; field: 19%). That may seem disappointing, considering the older pedestrians are the target group of the prototype. However, the older people participating in the two studies were all healthy and did not experience any problems themselves. Thus, it seemed that they did not consider themselves the real target group. That is supported by 59% of the participants of the field study saying they would recommend the system to people in need and 57% of the VR study saying that people would like the system, indicating an overall appreciation for the prototype. The likelihood of someone buying such a system seems to be related to their self-perception. This might be problematic as aging is a gradual process, and not always transparent to oneself. Therefore, in addition to the development of supporting technology, the awareness of potential risks must be raised in the older population.

### Comparison of VR study versus field test

Overall, the comparison of VR and field test results are in line with previous findings: the overall behavioral tendencies are the same (e.g., Schwebel et al., 2008). However, unlike in previous comparisons (Feldstein, 2019; Feldstein and Dyszak, 2020), the behavior in VR was not found to be riskier than in the real-world. In fact, the opposite is true, as the behavior in the VR, including stopping in the street was safer compared to the real-world behavior. Conversely, it can be argued that results are in line with the previous findings, as behavior in VR is more extreme than in the real-world. From our experience, we conclude that VR is a highly valuable way to do pedestrian research and research regarding the technical support of pedestrians. Especially for learning more about general behavioral patterns in a safe and controllable, but still very realistic, environment. Nonetheless, we consider the validation of results in the real-world as inevitable before drawing conclusions and giving suggestions.

### Limitations

There are several differences between the two experiments that reduce comparability of results.

The trigger mechanism for the two experiments was different. The vibration in the VR was triggerd at the distance of 2.25 m and was the same for each person in every trial. This distance was chosen for practical reasons to assure enough time to check for cars. In the field test, the system needed 1 s to analyse the data, detection of kerbstone took place at a distance of 2 m +/− 1 m from the street. Thus, the moment the vibration was given depended on the walking speed and the approaching angle of the person, which could vary between people and trials.

Reliability of the assistance systems used in the two experiments was different. The functioning of the system in the VR study was only simulated, thus a perfectly reliable system could be provided. In the field test, the system was actually working. It’s hit-rate was 0.922, which means it failed to generate vibration cues in 8% of the cases when people approached the street. Subjective trust was not assessed in the experiments. However, it is very likely that participants trusted the perfect system in the VR experiments more than the actual system.

Of course, the use of the walking frame is not ideal. Majority of the older population does not use a walking frame. All participants in the two experiments were healthy subjects not using any type of walking aid in their normal life. Thus, the walking experience in the two experiments was different from their normal walking behavior. It is also possible that the use of the walking frame did add additional workload to the situation. The use of the walking frame in the two experiments was necessary, because it had to carry the system (including a laptop) in the field test, and it was needed for safety reasons in the VR experiment. However, for a later stage of development of the assistance system, it is aimed to create a wearable device, that could be used without a walking frame in a field experiment and in reality.

The acceptance questions were chosen based on what seemed to be most suited for the respective experiment. However, two out of three questions differed between the experiments, which makes it difficult to compare the results. Data collection overlapped, which made it impossible to “learn” from the previous experiment for the next one.

It is important to mention that the walking situation was artificial, even in the field test, and that there are several differences between the two experiments. However, as both experiments are within-subjects designs, the potential impact of cars, surrounding, use of the walking frame, etc. were always present in both conditions. Thus, effects found for the assistance system are not confounded with anything else and can, therefore, be completely ascribed to the use of the system.

The two experiments represent two different ways of evaluation, using the benefits of the respective methods (VR vs. field). Even though the data is not comparable in a statistical manner, the joint description of the two different studies offers a lot of complementary information that allows for a solid overall evaluation of the assistance system.

### Practical implications

The assistance system that was evaluated is still in the state of a very early prototype. Albeit encouraging results, a lot of improvements are required. First of all, training of the detection algorithm has to be done again including the elements that caused false alarms (such as trees or other people). In the future course of further development, choice and placement of sensors should allow the use of a wearable device and to dispel the need for a walking frame. That would likely make the system appeal to a larger subset of the older population. Additionally, a long-term study has to be conducted in order to investigate whether positive behavioral changes during the use of the assistance system are stable.

Along with the development of technical assistance to support older pedestrians, awareness campaigns are needed. Older people should learn more about the underlying reasons for their higher risk during road crossings. Of course, it is very difficult to change behavioral patterns that have been practiced for years, but maybe the understanding of underlying mechanisms can help.

Active pedestrian assistance like the prototype that was evaluated in the current study is important alongside passive support through driver assistance targeting pedestrian protection. One approach that could close the gap between active and passive support is the P2C communication, which would offer both parties better options to avoid crashes.

### Conclusion

The prototype of the assistance system that has been developed by the FANS research group has been successfully evaluated in VR and the real-world. Findings are promising as the assistance system is able to increase the safety behavior of pedestrians in terms of checking for traffic. Furthermore, the system did not increase the subjective workload of participants, which could have been an unwanted side effect.

## Data availability statement

The raw data supporting the conclusions of this article will be made available by the authors, without undue reservation.

## Ethics statement

The studies involving human participants were reviewed and approved by Ethik-Kommission des Instituts für Psychologie und Arbeitswissenschaft (IPA) der TU Berlin. The patients/participants provided their written informed consent to participate in this study. Written informed consent was obtained from the individual(s) for the publication of any potentially identifiable images or data included in this article.

## Author contributions

RW was responsible for planning, conducting, and analyzing the experiment as well as for writing the article. JP was responsible for planning and conducting the experiment, and assisted with the analysis and the writing of the article. All authors contributed to the article and approved the submitted version.

## Funding

This research was funded by the Bundesministerium für Bildung und Forschung and the open access publication fee was funded by the Technische University Berlin.

## Conflict of interest

The authors declare that the research was conducted in the absence of any commercial or financial relationships that could be construed as a potential conflict of interest.

## Publisher’s note

All claims expressed in this article are solely those of the authors and do not necessarily represent those of their affiliated organizations, or those of the publisher, the editors and the reviewers. Any product that may be evaluated in this article, or claim that may be made by its manufacturer, is not guaranteed or endorsed by the publisher.

## Footnotes

^1^Wiczorek, R. (under review). Evaluation of thermotactile and vibrotactile cues to improve hazard perception of older pedestrians.

^2^Qureshi, H. S. (submitted). User-Centered Development of a Pedestrian Assistance System Using End-to-End Learning. Doctoral dissertation, Technische Universität Berlin.
